# Alleviation of Androgenetic Alopecia with Aqueous *Paeonia lactiflora* and *Poria cocos* Extract Intake through Suppressing the Steroid Hormone and Inflammatory Pathway

**DOI:** 10.3390/ph14111128

**Published:** 2021-11-05

**Authors:** Ting Zhang, Shihua Cao, Heng Yuan, Sunmin Park

**Affiliations:** 1 Department of Bio-Convergence System, Hoseo University, Asan 31499, Korea; zhangting92925@gmail.com (T.Z.); yuanheng.changan@gmail.com (H.Y.); 2 Obesity/Diabetes Research Center, Department of Food and Nutrition, Hoseo University, Asan 31499, Korea; caoshihua92@gmail.com

**Keywords:** androgenic alopecia, testosterone, *Paeonia lactiflora pall*, *Poria cocos*, inflammation, network pharmacology

## Abstract

*Paeonia lactiflora* Pallas (PL) and *Poria cocos* Wolf (PC) have been traditionally used to treat inflammatory diseases reported in Dongui Bogam and Shen Nong Ben Cao Jing, traditional medical books in Korean and China, respectively. We determined the efficacies and the molecular mechanisms of PL, PC, and PL + PC aqueous extracts on androgenetic alopecia (AGA) induced by testosterone propionate in C57BL/6 mice. The molecular mechanisms of PL and PC in AGA treatment were examined using experimental assays and network pharmacology. The AGA model was generated by topically applying 0.5% testosterone propionate in 70% ethanol solution to the backs of mice daily for 28 days while the normal-control (Normal-Con; no AGA induction) mice applied 70% ethanol. The 0.1% PL (AGA-PL), 0.1% PC (AGA-PC), 0.05% PL + 0.05% PC (AGA-MIX), and 0.1% cellulose (AGA-Con; control) were supplemented in a high-fat diet for 28 days in AGA-induced mice. Positive-control (AGA-Positive) were administered 2% finasteride daily on the backs of the AGA mice. Hair growth rates decreased in the order of AGA-PL, AGA-MIX, AGA-PC, AGA-Positive, and AGA-Con after 21 days of treatment (ED21). On ED28, skins were completely covered with hair in the AGA-PL and AGA-MIX groups. Serum testosterone concentrations were lower in the AGA-PL group than in the AGA-Con group and similar to concentrations in the Normal-Con group, whereas serum 17β-estradiol concentrations showed the opposite pattern with increasing aromatase mRNA expression (*p* < 0.05). In the dorsal skin, DKK1 and NR3C2 mRNA expressions were significantly lower, but TGF-β2, β-Catenin, and PPARG expressions were higher in the AGA-PL and AGA-PC groups than in the AGA-Con group (*p* < 0.05), whereas TNF-α and IL-6 mRNA expressions were lower in the AGA-PL, AGA-MIX, and Normal-Con groups than in the AGA-Con group (*p* < 0.05). The phosphorylation of Akt and GSK-3β in the dorsal skin was lower in AGA-Con than normal-Con, and PL and MIX ingestion suppressed their decrease similar to the Normal-Con. In conclusion, PL or PL + PC intake had beneficial effects on hair growth similar to Normal-Con. The promotion was related to lower serum testosterone concentrations and pro-inflammatory cytokine levels, and inhibition of the steroid hormone pathway, consistent with network pharmacology analysis findings.

## 1. Introduction

Androgenetic alopecia (AGA) is the most common cause of chronic progressive hair loss in young and middle-aged men [1] and has a worldwide incidence rate of about 20% [2]. AGA has a considerable negative impact on quality of life and physical and mental health [2]. The exact mechanism remains unclear, but excessive androgen receptor activation in the scalp shortens the anagen or growth phase in the hair growth cycle and miniaturizes the follicles. It contributes to thinner and shorter hair follicles, resulting in accelerating hair loss. Individuals lacking androgen receptors do not develop AGA [3]. AGA pathogenesis is also linked to endocrine factors and genetic susceptibility [4]. Inadequate oxygen and nutrient supply due to the reduced blood supply to the scalp may cause hair loss [3]. Inflammation, dyslipidemia, hyperglycemia, and insulin resistance are also involved in AGA etiology, and Western-style diets containing high sugar and cholesterol and a low mineral diet may increase the potential to induce AGA [5].

Topical minoxidil and finasteride for oral medication are clinically approved by the US Food and Drug Administration (FDA) and European Medicines Agency (EMA) for the treatment of AGA [6]. Finasteride is a 5α-reductase inhibitor to suppress dihydrotestosterone production and reverses male hair loss, although its efficacy highly varies between individuals [7]. However, they have safety issues, and the overall successful treatment rates are low [8]. Topical finasteride has been examined for AGA treatment with decreasing complications induced with oral administration [9]. Topical finasteride has a phase III clinical trial, demonstrating the increase of hair count and decreased adverse effect compared to placebo [10]. A systematic review of topical finasteride efficacy for AGA demonstrates a positive result for AGA treatment (0.1–1%), with a favorable safety result in men [9]. Topical finasteride (2%) is reported to increase follicular density without increasing serum finasteride in male hairless animals with topical testosterone solution [11,12]. Researchers have turned to Traditional Chinese Medicine (TCM) as a source of potential AGA medication in recent years [13].

*Paeonia lactiflora* Pallas (PL; English name: peony) has been used as traditional Chinese medicine (TCM) reported in the classic Ming prescription “Wu Tou Decoction” from Jin Gui Yao Lue [14] and also “Donguibogam” from Lee’s Chosun Dynasty in Korea (Jun, 1613). In “Gu Tu Qi Fang”, *Paeonia lactiflora* Pallas is recorded to replenish the essence of blood, stabilize hair loss, and promote hair growth in persons with hair loss due to deficient essence of blood (Qi, 2019). It also reduces inflammation and pain and improves blood circulation and hyperglycemia [15]. Paeoniflorin, peony glycosides, and 8-debenzoylpaeoniflorin, the PL components, have been reported to have immune regulation and anti-inflammatory activity [16] and reduce cerebral hemorrhage [17]. Meanwhile, *Poria cocos* Wolf (PC) can be traced back to an ancient Chinese medical book “Shen Nong Ben Cao Jing” [18] and “Huang Di Nei Jing” (Liang et al., 2014), where it is reported to have been used for edema, nephrosis, and chronic gastritis, and relieve mental stress. The chemical constituents of PC mainly include triterpenes, polysaccharides, and steroids. Triterpenoids in PC have been reported to have beneficial impacts on autoimmune-related diseases [19] and hypoglycemic [20], whereas polysaccharides of PC promote immune response [21]. Since AGA is partially related to the immune privilege collapse in the hair follicles and hyperglycemia, the suppressors of pro-inflammatory and hyperglycemia activities are usually administered to treat the condition [5,22], which suggests PL and PC might alleviate AGA symptoms by promoting anti-inflammatory activities.

The effectiveness of medications can be screened by network pharmacology before conducting experimental studies. Network pharmacology has recently been used to check the effectiveness and mechanisms responsible for TCM on various diseases and provide potential action mechanisms [23]. The chemical components of PL and PC are retrieved from the TCM system pharmacology database and analysis platform (TCMSP) and their active ingredients and related targets of AGA are screened. Mice hair follicles express androgen receptors, which testosterone binds to and activates to inhibit hair growth, leading to hair loss [24,25,26]. We preliminarily confirmed the hair loss in mice induced by applying testosterone propionate solution into the dorsal skins of C57BL/6, commonly used as an animal model for studying the efficacy and mechanism of hair growth according to the hair follicle growth cycle [25,27]. This is a similar etiology of human AGA and this was used as an animal model in the present study. Topical application of 2% finasteride solution was used as a positive control in previous and present studies [25]. We aimed to evaluate the efficacy of PL and PC in AGA treatment and their molecular mechanism in network pharmacology and an established AGA animal model. We hypothesized that PL and PC intake might improve hair growth in the AGA animal model by suppressing testosterone-related pathways because of their suppressive effects on androgen, hyperglycemia, and pro-inflammatory cytokines. The hypothesis was examined in C57BL/6 mice with testosterone-induced alopecia [25,26]. This study was novel to show the PL and PL + PC intake had a potential AGA treatment in AGA animal model through modulating lowering inflammation and steroid hormone pathway and network pharmacology analysis supported the results.

## 2. Results

### 2.1. Screening of Active Ingredients in PL and PC and Predictions of Potential Targets for AGA Treatment Using TCMSP

Using OB (≥30%) and DL (≥0.18) as required by the TCMSP database [23,28], 13 and 15 relevant active ingredients of PL and PC, respectively, were identified (Table 1). Paeoniflorgenone, paeoniflorin, albiflorin, and 12beta-olide of PL had the highest OB (>60%) and DL (0.3–0.8) values, whereas dehydroeburicoic acid, ergosta-7, 22E-dien-3beta-ol, ergosterol peroxide, pachymic acid, and poricoic acids of PC had the highest OB (30–40%) and DL (0.7–0.8) values (Supplemental Table S1).

In total, 48 and 14 molecular targets of active ingredients of PL and PC, respectively, and 3412 and 3431 molecular targets of PL and PC, respectively, on AGA, were also collected. Twenty-three and four potential targets for PL and PC, respectively, intersected with AGA targets. They were used for TCMSP analysis.

### 2.2. Network Topology Diagram of “Active Component-Disease-Target” for PL and PC

**Figure 1:**
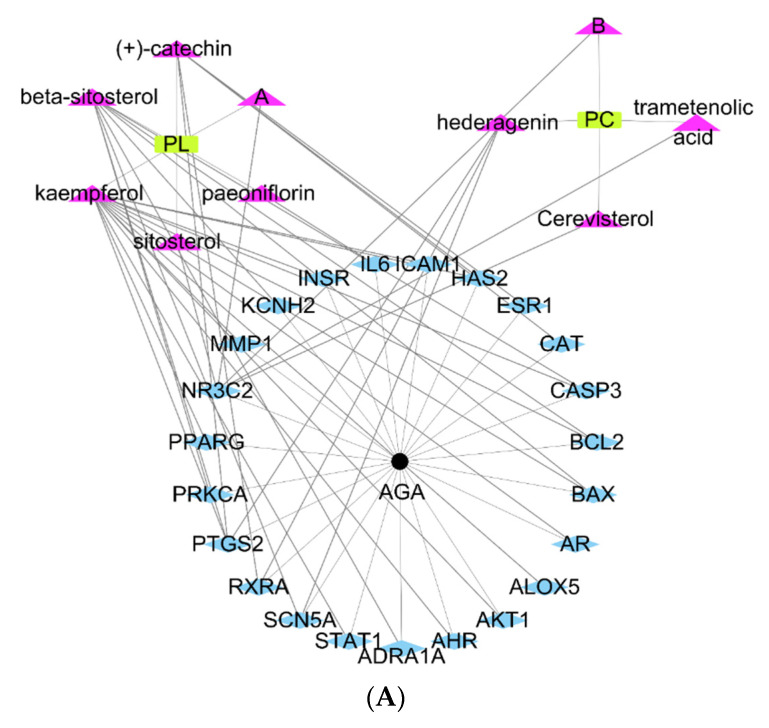

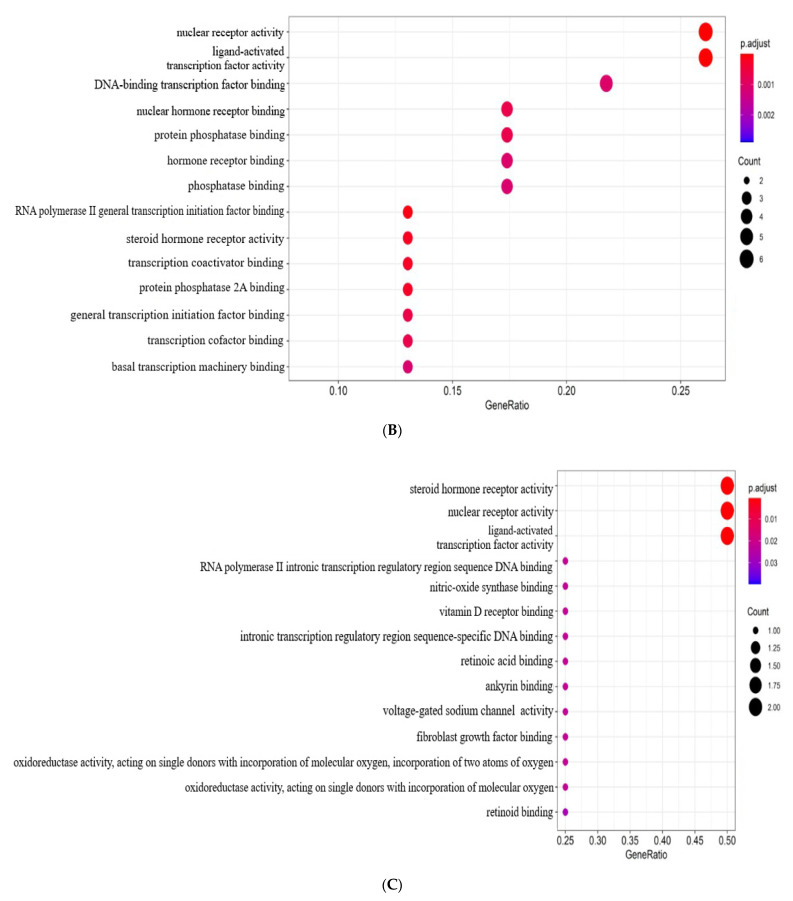
Active component-target network and gene ontology (GO) function enrichment analysis of active components of *Paeonia Lactiflora (PL)*, *Poria Cocos (PC)*, and androgenetic alopecia (AGA). (**A**) Active component-target network of primary active ingredients of *Paeonia Lactiflora* (PL) and *Poria Cocos* (PC). (**B**) GO function enrichment analysis results between PL and AGA. (**C**) GO function enrichment analysis results between PC and AGA. Main active ingredients (triangle-purple), targets (rhombus-blue), PC and PL (rectangle-yellow), AGA (dots-black). The X-axis represents the number of genes enriched in each function, and the Y-axis is the name of the function. Different colors represent different degrees of enrichment. The redder color and the bigger bubble indicated a higher degree of enrichment of the target pathways and a smaller *p*-value.

The network topology diagram of “Active-component-disease-target” showed interactions between active ingredients of PL and PC and AGA treatment (Figure 1A). In Figure 1A, interactions that involved 23 target genes are shown as blue rhombuses, AGA is represented as black dots, located in the middle of the target genes, and the active compounds of PC and PL are shown as pink triangles. PL and PC had six and four core active ingredients for AGA treatment, respectively.

### 2.3. PPI Network Analysis and GO Enrichment Analysis

PPI network analysis revealed 20 protein nodes and 85 edges (connection degrees) with *AKT1* as the core gene and the presence of protein–protein interactions (Supplemental Figure S1).

The functional distribution of PL and PC prediction targets was explored by GO analysis. The X-axis represents the number of genes enriched in each function, and the Y-axis represents the function related to active components of PL and PC (Figure 1B). Different colors represent different degrees of enrichment and statistical differences: the darker red the color, the greater the enrichment degree (Figure 1B). GO analysis showed that the predicted PL targets were enriched in the mechanisms of nuclear receptor activity, ligand-activated transcription factor activity, DNA-binding transcription factor binding, nuclear hormone receptor binding, and protein phosphatase binding (*p* < 0.001; Figure 1C). The predicted molecular targets for PC were mainly enriched steroid hormone receptor activity, nuclear receptor activity, and ligand-activated transcription factor activity (*p* < 0.01; Figure 1C).

### 2.4. Contents of Index Compounds in PL and PC, Food and Herbal Intake, Body Weight, and Fat Weight in Mice

PL contained 54.01 ± 2.70 mg paeoniflorin/g dry powder, while PC contained 0.56 mg ± 0.02 mg pachymic acid (Supplemental Figure S2).

**Figure 2:**
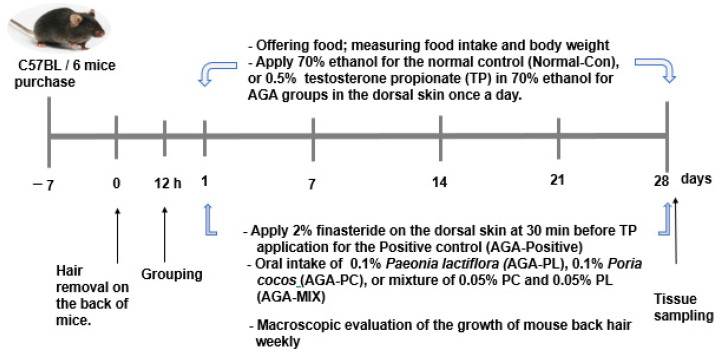
Experimental design of the animal study. AGA was induced with the application of 0.5% testosterone propionate solution on the back of mice, and AGA-induced mice orally consumed 0.1% PL, 0.1% PC, or 0.1% mixture of PL and PC (1:1, *w*/*w*) in a 43% fat diet (HFD), called AGA-PL, AGA-PC, or AGA-MIX groups, respectively. AGA-induced mice were daily applied with 2% finasteride on the back, as AGA-Positive (positive control). AGA-Positive and AGA-Con (control) mice had 0.1% cellulose (no effective activity in AGA treatment) in an HFD. Normal-Con did not induce AGA and consumed 0.1% cellulose in an HFD. All mice had an 8-week treatment.

The experimental schedule is presented in Figure 2. After a 28-day intervention, the mean final weight tended to be lower in the AGA-PC and AGA-MIX groups than in the other groups, but not significantly (Table 1). However, weight gain in the AGA-PC and AGA-MIX groups was lower than in the AGA-Positive group (*p* < 0.05) (Table 1). Although no significant difference in food intake was observed among groups, food efficiency in the AGA-MIX group was lowest, and food efficiencies were similar in the AGA-PL, AGA-PC, and AGA-Con groups (Table 1). PL and PC ingestion were not significantly different in the AGA-PL and AGA-PC groups (about 147 mg/kg/day, Table 1). PL and PC mixture ingestion lowered epididymal fat, retroperitoneal fat, and total visceral fat contents than AGA-Con mice, while they were lowest in the AGA-Positive group among all groups (*p* < 0.05) (Table 1).

### 2.5. Hair Growth Status and Clinical Hair Growth Scores

**Figure 3:**
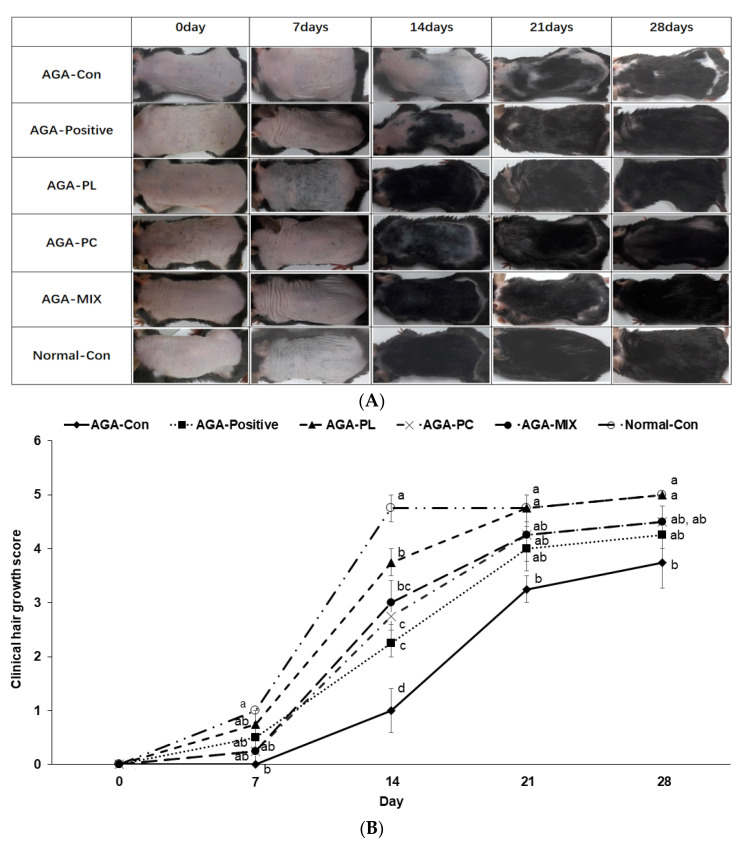

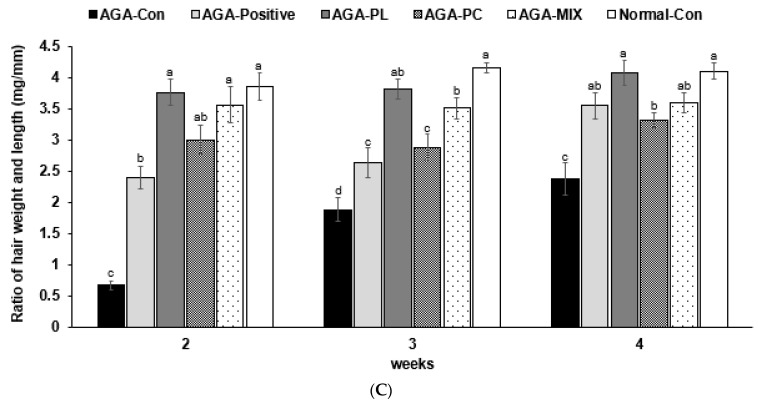
Severities of AGA symptoms. (**A**) Changes in AGA symptoms of dorsal skin lesions between treatment start and finish on intervention day 28. (**B**) Evaluation of average hair growth statuses of clinical AGA symptoms from treatment start to finish. (**C**) The ratio of hair weight to the length of the 30 hairs on the 2nd, 3rd, and 4th week. AGA was induced with the application of 0.5% testosterone propionate solution on the back of mice, and AGA-induced mice orally consumed 0.1% PL, 0.1% PC, or 0.1% mixture of PL and PC (1:1, *w*/*w*) in a 43% fat diet (HFD), called AGA-PL, AGA-PC, or AGA-MIX groups, respectively. AGA-induced mice were daily applied with 2% finasteride on the back, as AGA-Positive (positive control). AGA-Positive and AGA-Con (control) mice had 0.1% cellulose (no effective activity in AGA treatment) in an HFD. Normal-Con did not induce AGA and consumed 0.1% cellulose in an HFD. All mice had an 8-week treatment. Bars and error bars represented means and standard deviations (N = 11 or 12). ^a,b,c,d^ Different letters on the bars indicate a significant difference as per a Tukey test at *p* < 0.05, and no or the same letters indicate no significance between the groups.

On experimental day 7, as many new hairs were visible in the AGA-PL group as the Normal-Con group (Figure 3A). On experimental day 14, PL and PC ingestion was associated with fast hair growth, but hair growth was just initiated in the AGA-Con group. At this time, hair growth in the AGA-Con was observed on less than 20% of the exposed dorsal skin but more than 20% in the AGA-Positive and AGA-PC groups, and more than 80% in the AGA-PL, AGA-MIX, and Normal-Con groups (Figure 3A,B). On experimental day 21, exposed skin was visible in the AGA-Con group, while dorsal skin was completely covered with as much hair in the AGA-PL group as in the Normal-Con group, but slightly exposed in the AGA-Positive, AGA-MIX, and AGA-PC groups (Figure 3A,B). On experimental day 28, most dorsal skin tissues were still exposed in the AGA-Con group, partially exposed in the AGA-PC group, slightly exposed in the AGA-Positive and AGA-MIX groups, and completely covered in the AGA-PL and the Normal-Con groups (*p* < 0.05) (Figure 3A,B). Overall, mice in the AGA-PL and AGA-MIX groups exhibited faster skin hair growth than those in the AGA-Con group (*p* < 0.05).

The result of the 1st week was not added to Figure 3C, since the hair on the back did not grow in any group. In the 2nd week, the ratio of hair length and weight was significantly higher in the treatment groups than in the AGA-Con group, and the ratio of the AGA-PL and AGA-MIX groups was similar to that of Normal-Con (*p* < 0.05; Figure 3C). In the 3rd and 4th weeks, the hair length ratio with weight was higher in the treatment groups than that of the AGA-Con group. AGA-PL increased the ratio as much as the Normal-Con (*p* < 0.05; Figure 3C). PL and PL and PC mixture ingestion was associated with complete hair growth in AGA mice during the 8-week treatment.

### 2.6. Histopathology of Dorsal Skins

**Figure 4:**
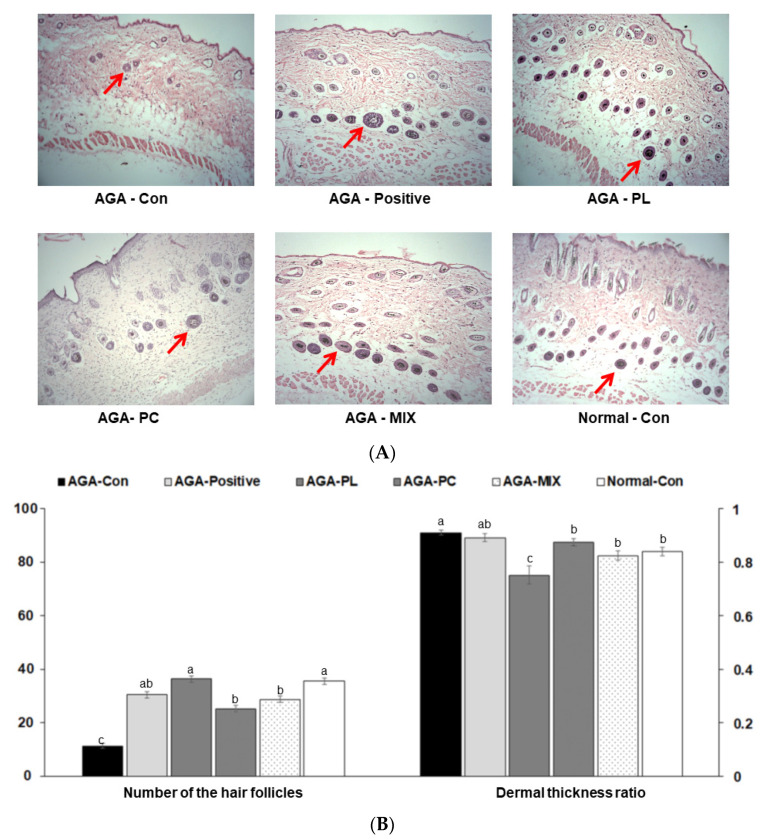
Histological differences and hair follicle densities in dorsal skins. (**A**) Histology of H&E-stained dorsal skins. (**B**) The red arrows indicate the hair follicle. AGA was induced with the application of 0.5% testosterone propionate solution on the back of mice, and AGA-induced mice orally consumed 0.1% PL, 0.1% PC, or 0.1% mixture of PL and PC (1:1, *w*/*w*) in a 43% fat diet (HFD), called AGA-PL, AGA-PC, or AGA-MIX groups, respectively. AGA-induced mice were daily applied with 2% finasteride on the back, as AGA-Positive (positive control). AGA-Positive and AGA-Con (control) mice had 0.1% cellulose (no effective activity in AGA treatment) in HFD. Normal-Con did not induce AGA and consumed 0.1% cellulose in HFD. All mice had an 8-week treatment. After 28 days of treatment, dorsal skins were fixed with 10% formaldehyde, embedded in paraffin, sectioned, and stained with hematoxylin and eosin. Original magnification 100×. Bars and error bars are mean ± standard deviations (N = 11 or 12). ^a,b,c^ Different letters on the bars indicate a significant difference as per a Tukey test at *p* < 0.05, and no or the same letters indicate no significance between the groups.

On experimental day 28, hair follicles were miniaturized, and hair densities were significantly lower in the AGA-Con group than in the other groups (Figure 4A,B). Mice in the Normal-Con, AGA-PL, AGA-PC, and AGA-MIX groups had significantly greater hair follicle densities than those in the AGA-Con group (*p* < 0.05) (Figure 4A,B). The number of hair follicle was similar in the AGA-MIX and AGA-Positive groups. Mice in the AGA-Con groups had significantly greater dermal thickness than those in the AGA-PL and AGA-MIX group (*p* < 0.05) (Figure 4A,B). The dermal thickness ratio was similar in the AGA-Positive, AGA-PC, and Normal-Con groups (Figure 4B). These results suggested that PL ingestion exhibited more effectiveness for AGA therapy than PC and PC and PL mixture ingestion. PL ingestion was associated with promoting hair growth as much as finasteride.

### 2.7. Serum Testosterone, 17β-Estradiol, and Triglyceride Concentrations

After the intervention, serum testosterone concentrations were lowered only in the AGA-PL group, and were similar to those in the Normal-Con (*p* < 0.05) (Table 2). AGA-PC and AGA-MIX tended to lower serum testosterone concentrations, but these reductions were not significant. Serum 17β-estradiol concentrations were higher in the Normal-Con and AGA-PL groups than in the AGA-CON group (Table 2), but lower in the AGA-Positive, AGA-PC, and AGA-MIX groups. Serum triglyceride concentrations were lower in the AGA-PC and AGA-PL groups than in the AGA-CON and similar to those observed in the Normal-Con group (*p* < 0.05) (Table 2).

### 2.8. mRNA Expressions of AGA-Related Genes in Dorsal Skin

**Figure 5:**
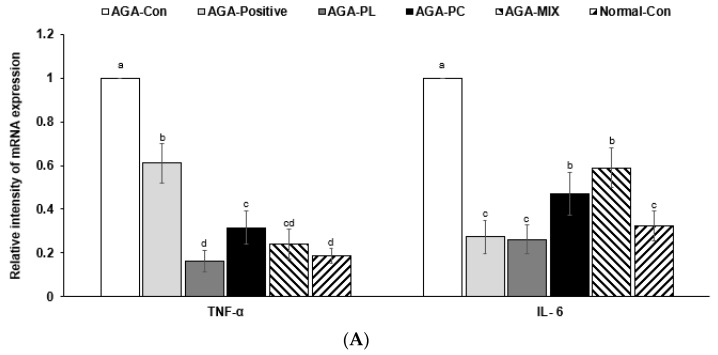

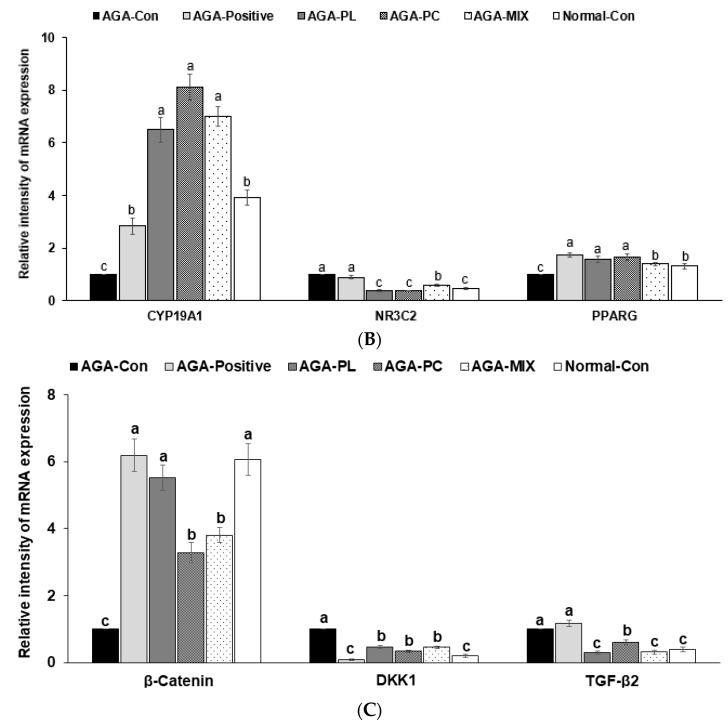
mRNA expression levels in the dorsal skin. (**A**) Tumor necrosis factor-α (*TNF-α*) and interleukin 6 (*IL-6*). (**B**) Aromatase (*CYP19A1*), nuclear receptor subfamily 3 group (*NR3C2*), and peroxisome proliferator-activated receptor gamma (*PPARG*). (**C**) *β-Catenin*, Dickkopf-related protein 1 (*DKK1*), and tumor growth factor-β2 (*TGF-β2*). Bars and error bars are mean ± standard deviations (N = 4). ^a,b,c^ Different letters on the bars indicate a significant difference as per a Tukey test at *p* < 0.05, and no or the same letters indicate no significance between the groups. AGA was induced with the application of 0.5% testosterone propionate solution on the back of mice, and AGA-induced mice orally consumed 0.1% PL, 0.1% PC, or 0.1% mixture of PL and PC (1:1, *w*/*w*) in a 43% fat diet (HFD), called AGA-PL, AGA-PC, or AGA-MIX groups, respectively. AGA-induced mice were daily applied with 2% finasteride on the back, as AGA-Positive (positive control). AGA-Positive and AGA-Con (control) mice had 0.1% cellulose (no effective activity in AGA treatment) in an HFD. Normal-Con did not induce AGA and consumed 0.1% cellulose in an HFD. All mice had an 8-week treatment.

After the intervention, the mRNA expressions of tumor necrosis factor-α (*TNF-α*) and interleukin 6 (*IL-6*) cytokines in dorsal skin tissues were much higher in the AGA-Con group than in the Normal-Con group (Figure 5A), but similar in the AGA-PL and Normal-Con groups, and also reduced in the AGA-PC and AGA-MIX groups, but not as much as that observed in the AGA-PL group. Their expressions were also reduced in the AGA-Positive group compared with AGA-Con, but the decrease of *IL-6* mRNA expression was much more significant than that of *TNF-α* (Figure 5A).

Nuclear receptor subfamily 3 Group (*NR3C2*) and peroxisome proliferator-activated receptor gamma (*PPARG*) genes are related to steroid hormone receptor signaling, and their expressions are negatively related to hair growth. After the intervention, *NR3C2* mRNA expression was highest in the AGA-Con group (Figure 5B), but similar in the AGA-PL, AGA-PC, AGA-MIX, and Normal-Con groups. Interestingly, its expression was similar in the Positive-Control and AGA-Con groups. On experimental day 28, group *PPARG* mRNA expressions were lowest in the AGA-MIX and Normal-C groups and similar in the AGA-PL, AGA-PC, and Positive-Control groups (Figure 5B).

Aromatase (CYP19A1) converts androgens into estrogen in scalp hair follicles. The increase in estrogen in the experiment promotes hair growth. *CYP19A1* mRNA expression was much lower in the AGA-Con than in the AGA-PL, AGA-PC, and AGA-MIX groups (Figure 5B). *CYP19A1* mRNA expression was markedly and similarly lower in AGA-Positive and AGA-Con groups (Figure 5B).

Hair growth is also associated with Wnt signaling: Dickkopf Wnt signaling pathway inhibitor 1 (*DKK1*) and catenin beta-1 (*β-catenin)* are the central regulators of hair growth. TGF-β signaling interacts with Wnt signaling. The mRNA expression of *DKK1,* an inhibitor of the Wnt signaling pathway, was much higher in the AGA-Con group than in the other groups (Figure 5C). AGA-PL, AGA-PC, and AGA-MIX also reduced *DKK1* expression but not to the same extent observed in the AGA-Positive group (Figure 5C). The mRNA expression of *β-catenin*, an indicator of Wnt signaling activation, was much lower in the AGA-Con than in the Normal-Con, AGA-Positive, and AGA-PL groups. PC and PL/PL also reduced its expression but not to the same extent as AGA-PL (Figure 5C). In addition, *TGF-β2* mRNA expression was much higher in the AGA-Positive and AGA-Con group than in the other groups (Figure 5C). *TGF-β2* mRNA expression was markedly lower in AGA-PL, AGA-MIX, and Normal-Con groups than AGA-Con (Figure 5C). These observations suggested that finasteride acted on Wnt signaling to promote hair growth, but PL ingestion promoted aromatase and Wnt signaling pathways.

### 2.9. Phosphorylation of Akt and GSK-3β in the Dorsal Skin

**Figure 6:**
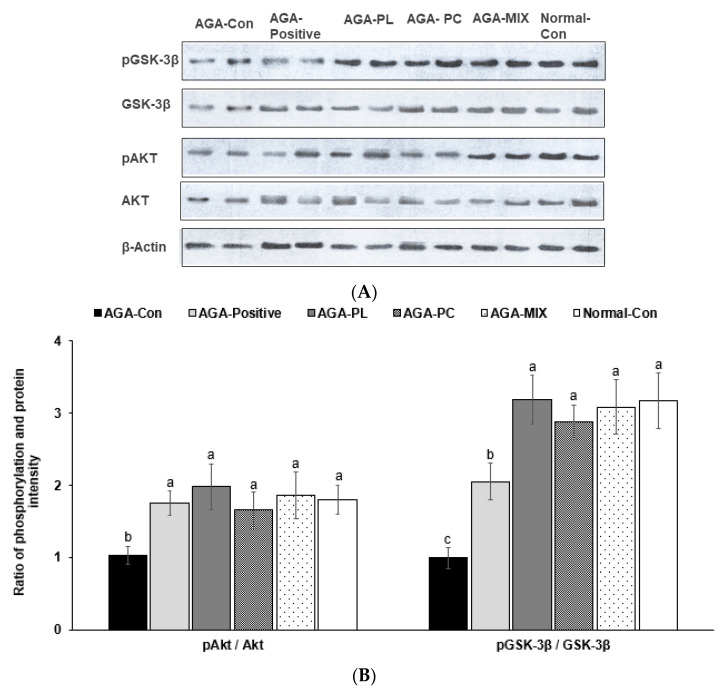
Phosphorylation of Akt and GSK-3β in the dorsal skin. (**A**) The phosphorylation and protein contents of Akt and glycogen-synthase kinase-3β (GSK-3β). (**B**) The ratio of the phosphorylation and protein of Akt and GSK-3β. Bars and error bars are mean ± standard deviations (N = 4). ^a,b,c^ Different letters on the bars indicate a significant difference as per a Tukey test at *p* < 0.05, and no or the same letters indicate no significance between the groups. The insulin signaling pathways were measured with western blotting analysis, and an image analyzer measured their intensity. AGA was induced with the application of 0.5% testosterone propionate solution on the back of mice, and AGA-induced mice orally consumed 0.1% PL, 0.1% PC, or 0.1% mixture of PL and PC (1:1, *w*/*w*) in a 43% fat diet (HFD), called AGA-PL, AGA-PC, or AGA-MIX groups, respectively. AGA-induced mice were daily applied with 2% finasteride on the back, as AGA-Positive (positive control). AGA-Positive and AGA-Con (control) mice had 0.1% cellulose (no effective activity in AGA treatment) in an HFD. Normal-Con did not induce AGA and consumed 0.1% cellulose in an HFD. All mice had an 8-week treatment.

Akt and GSK-3β phosphorylation are involved in Wnt signaling to promote hair growth. Phosphorylation of Akt and GSK-3β to their proteins was significantly higher in the AGA-PL, AGA-MIX, and Normal-Con groups than the AGA-Con (*p* < 0.05; Figure 6A,B). The increase of GSK-3β phosphorylation was higher in the AGA-Positive group, but its increase was much lower than the other treatment groups (Figure 6A,B).

## 3. Discussion

We undertook this study to determine whether PL and PC intake would improve hair growth in AGA-induced C57BL/6 mice and reduce androgen and pro-inflammatory cytokine contents. Before the animal experiment, the efficacy and action mechanisms of PL and PC were evaluated by system pharmacology network analysis [23], which showed their potential efficacies are derived mainly by their effects on steroid hormone receptor activity, nuclear receptor activity, and ligand-activated transcription factor activity. It indicated that PL and PC had the potential to alleviate AGA and their efficacy and mechanism were examined in a murine animal model. PL and PC increased hair follicle densities by reducing serum testosterone concentrations and pro-inflammatory concentrations. Its mechanism was validated to increase the mRNA expression of aromatase and nuclear receptor expression to promote Wnt-related signaling in the dorsal skin. As a result, PL and PC protected against hair loss, and PL had a better protection effect on AGA.

AGA causes the progressive miniaturization of hair follicles and induces androgen-like effects in the epithelial cells of hair follicles in androgen-dependent regions [4,29]. The androgens secreted by the human body combine with type 2 5α-reductase in hair follicles to form DHT, which attacks hair follicles, causing them to shrink, which weakens hair strength, and eventually causes hair loss [30,31]. Aromatase reduces testosterone to inhibit testosterone-related hair loss [32]. We observed that AGA-CON mice had lower hair follicle densities and sizes and that PL, PL/PC, and finasteride protected against these effects. PL and PC have usually been reported to have anti-inflammatory effects in various inflammation-associated diseases [33,34]. Ong et al. [35] showed that paeoniflorin, a dominant component of PL, suppresses the over-secretion of testosterone induced by dexamethasone in theca cells by downregulating *CYP17A1* (aromatase) and *CYP11A1* and suggested PL might prevent excessive testosterone levels by converting to estrogen. No study has been previously conducted on the effects of PC or PL on AGA. The present study consistently showed that PL and PC increased *CYP17A1 mRNA* expression related to converting testosterone into estrogen and both elevated serum 17β-estradiol concentration. Thus, PL and PC can be a candidate of therapeutic agents for AGA, although the pathway to treat AGA may be different from finasteride.

Improvements in living standards have increased the desire for AGA treatment, but safer, more effective drugs are required. TCM provides treatments with multiple components that target many bio-entities [36]. Integrating with the recently developed network pharmacology aids understanding of the systemic therapeutic mechanisms of TCMs [37]. Network pharmacology can be used to investigate the effectiveness and action mechanisms of TCMs based on their active ingredients and disease targets [23,28]. In the present study, we conducted an integrated analysis of AGA treatment by PL and PC and investigated the interactions between their active components and genes related to AGA. The network pharmacology data provided the PL and PC efficacy on AGA and their potential side effects of strengthening the feasibility of the animal study results.

Paeoniflorin, albiflorin, penta-O-galloyl-β-d-glucose, and other ingredients of PL have anti-oxidant, anti-inflammatory, anti-viral, and anti-tumor activities [15]. Polysaccharides in PC extract have been shown to possess potent anti-tumor and anti-apoptotic activities and immune regulatory, hypoglycemic, anti-inflammatory, and anti-oxidant effects [38,39]. System network pharmacology analysis indicated that PL might be an effective treatment for AGA and that PC might also be effective but not as effective as PL. However, system biology analysis did not support that combined treatment of PL and PC in AGA might be effective.

Serum androgen levels are elevated in AGA [30,40]. In this study, after topical application of testosterone propionate solution, the dorsal hair growth by C57BL/6 mice was inhibited by disturbing the synchronized growth cycle at the initial stage [40]. Changes in the hair growth cycle resulted in skin color changes from fleshy pink to gray and black, as previously described [30,41]. In the present study, after C57BL/6 mice had ingested PL, hair growth in dorsal skin was quickly started, and the numbers and sizes of hair follicles increased, suggesting that hair quickly entered the growth phase. Hair growth in the AGA-PC group was slower initially but faster during the later stage. Hair growth in the AGA-MIX and AGA-Positive groups were similar. These results show that PL and PL/PC reduced the symptoms of AGA in our murine model.

AGA is positively associated with immune modulation and inflammation, and thus, anti-inflammatory agents can be used to treat the condition [42]. In previous studies, PC was found to reduce the mRNA expressions of *TNF-α* and *IL-6* and to combat chronic nonbacterial prostatitis (a male disease) and arteriosclerosis [39,43]. Chronic nonbacterial prostatitis can be relieved by regulating testosterone, DHT, and 17β-estradiol levels [43]. Interestingly, in the present study, PL significantly reduced serum testosterone and elevated 17β-estradiol levels, reduced the mRNA expressions of pro-inflammatory cytokines (*TNF-α* and *IL-6*)*,* and lowered *NR3C2* levels in the AGA-PL group than in the AGA-PC group (*p* < 0.05). Thus, PL more effectively inhibited pro-inflammatory cytokines [44]. We also found that AGA-Con reduced the *PPARG* expression and the other treatment groups raised the expression of *PPARG*. The potentiation of Akt and GSK-3β phosphorylation improved Wnt signaling to protect against hair loss induced by testosterone. Finasteride provides a recognized treatment for AGA [6,41,45] and has also been used to treat prostate cancer [46]. We demonstrated that finasteride had no apparent effect on hair growth during the early stage of the experiment, but that subsequently, it quickly promoted hair growth.

In conclusion, PL ingestion effectively promoted hair regeneration with increased hair follicle numbers and sizes in our C57BL/6 mouse model of AGA by reducing serum testosterone and pro-inflammatory cytokines levels and steroid nuclear receptor expression and promoting the expressions of aromatase and Wnt-related transcription factors. System pharmacology analysis showed that the active ingredients of PC and PL also modulated steroid hormone receptor activity, nuclear receptor activity, and ligand-activated transcription factor activity, and these results were consistent with our animal experimental results. In clinical symptoms of hair loss, PL exhibited better protection against hair loss than PC. We concluded that PC and mixtures of PC and PL are potential treatments for AGA in an AGA murine model. This needs to be confirmed in a human study in the future.

## 4. Materials and methods

### 4.1. Screening PL and PC Active Ingredients as Potential Medications

The TCMSP contains 499 Chinese medications, 29,384 TCM-related components, 3311 targets, 837 associated diseases [23], and metabolism characteristics, including absorption, distribution, metabolism, and excretion (ADME), of each component. Drug screening criteria oral bioavailability (OB) and drug-likeness (DL) were set at ≥30% and ≥0.18, respectively [28]. OB is defined as the percentage of unchanged drug that reaches the systemic circulation after oral intake, whereas DL is quantified by scoring “drug-like” properties to optimize pharmacokinetics and pharmaceutical characteristics, such as solubility and stability. We searched all chemical components contained in “bai shao”, “*Paeonia lactiflora* Pallas”, “fu ling”, and “*Poria cocos* Wolf” in the TCMSP. Active ingredients of PL and PC were selected for a network topology using the drug-related target prediction function in TCMSP [47].

### 4.2. A Network Topology Map of PL and PC Bioactive Ingredient Targets and AGA Targets

Potentially related targets of PL and PC bioactive components were screened from the TCMSP database (https://tcmsp-e.com, accessed on 4 March 2020) [47] and they were converted into canonical SMILES standard format using PubChem database (https://pubchem.ncbi.nlm.nih.gov/, accessed on 12 March 2020). The converted PL and PC active compounds were imported into the Swiss Target Prediction database (http://www.swisstargetprediction.ch/, accessed on 19 March 2020). The molecular targets of active components of PL and PC were collected. After the conversion of their target names into official gene symbols using the Uniprot database (https://uniprot.org/, accessed on 26 March 2020), duplicated targets and those with no corresponding gene names were deleted. The targets of “androgenetic alopecia” were searched in the Gene cards (https://www.genecards.org/, accessed on 31 March 2020) [48], DisGeNET (https://www.disgenet.org/home/, accessed on 16 April 2020), NCBI gene (https://www.ncbi.nlm.nih.gov/gene, accessed on 24 April 2020), OMIM (https://omim.org/, accessed on 6 May 2020) [49], and Mala Cards databases (https://www.malacards.org, accessed on 20 May 2020) [50] to identify disease-related targets, and duplicate gene targets were deleted. Human genome database using “androgenetic alopecia”, “seborrheic alopecia”, and “hair loss” as keywords. The targets of the active component of PL and PC and related targets of AGA were integrated into unified UNIPROT ID through the Uniport database (https://www.uniprot.org/, accessed on 25 May 2020) and mapped with VENNY2.1 (https://bioinfogp.cnb.csic.es/tools/venny/index.html, accessed on 29 May 2020). To identify disease-related targets, and duplicate gene targets were deleted. Finally, the potential targets of active components and AGA were matched, and common targets were selected. This target data were imported into Cytoscape 3.8.2 software (https://www.cytoscape.org, accessed on 1 June 2020) to construct the “drug-component-disease-target” network topology. In the network, nodes represented active ingredients, target genes, and disease targets. The principle of node connection was to use edges to represent active ingredients and target genes and relationships between AGA and target genes. The multi-components, multi-targets, and multi-pathways of PL and PC against AGA therapy were examined by constructing a network topology diagram.

### 4.3. Protein–Protein Interaction (PPI) Network Construction and Gene Ontology (GO) Enrichment Analysis

The potential targets of PL and PC for AGA treatment were input into the STRING database (https://www.stringdb/, accessed on 4 June 2020) [51]. When constructing a PPI network for the active ingredients of PL and PC for AGA treatment, the organism was set for homo sapiens. Data received from the STRING database were sorted and imported into Cytoscape 3.8.2, and summary statistics of the network were analyzed using the “Network Analyzer” function of the program. Key targets with higher degree values than the average values were selected.

Drug-disease intersection (overlapping) targets were obtained, as described in Section 2.2, and were introduced into the *Kyoto Encyclopedia of Genes and Genome* (KEGG) database generated from Kanehisa Laboratories, Kyoto University (Kyoto, Japan) (https://www.kegg.jp/, accessed on 11 June 2020) to extract a canonical pathway highly related to these target proteins. Potential therapeutics among PL and PC active components with different molecular functions related to AGA were identified by GO enrichment analysis using the Bioconductor package (http://www.bioconductor.org/, accessed on 24 June 2020) [52]. The Bioconductor database (http://www.bioconductor.org/, accessed on 30 June 2020) analyzed biological processes, cellular components, and molecular functions of potential targets by PL and PC. The enrichment analysis of PL-AGA and PC-AGA by KEGG and GO was determined with their action molecular pathways. Statistical significance was examined at *p* < 0.05 with the R program.

### 4.4. Preparation of PL and PC Water Extracts and Analysis of Their Index Compounds

The root of PL and the sclerotium of PC were purchased from Kyungdong Herbal Market (Seoul). Dr. Young Seng Joo at Woo Suk University confirmed their authenticity in 2019, and it was stored in Hoseo University. Both were boiled in water at 95 ℃ for 3 h, filtered, and filtrates were evaporated in a rotary evaporator to 25% (*w*/*v*). The concentrates were lyophilized with a freeze dryer (IlShinBioBase, Dongdoocheon, Korea). The yields of PL and PC were 17.4 and 21.5%, respectively.

The index compounds of PL and PC were paeoniflorin and pachymic acid, respectively. Their contents in the extracts were measured using HPLC analysis. Quantitative analysis was conducted by peak integration using external paeoniflorin and pachymic acid standards having ≥95% purity purchased from Tokyo Chemical Industry Co., Ltd. (Tokyo, Japan). The HPLC condition was provided in the legend of Supplemental Figure S1.

### 4.5. Experimental Animal Model

Sixty 6-week-old male C57BL/6 mice (20 g–26 g) were purchased from Dae Han Bio Link (Um-Sung, Korea). Animals were adapted for one week in an animal facility in separate cages under controlled conditions (temperature: 20 ± 2°C, humidity: 65 ± 5%) under a 12-h light/dark cycle (08:00–20:00 h/20:00–08:00 h) and provided food and water ad libitum. The study protocols complied with the guidelines issued by the National Institute of Health and Animal Care and were approved beforehand by the Animal Care and Use Review Committee of Hoseo University (Asan, Korea; HSIACUC-18-230; approved on 6 January 2019).

After anesthetizing C57BL/6 mice with an intramuscular injection of a mixture of ketamine and xylazine (100 and 10 mg/kg of body weight, respectively; Bayer AG, Leverkusen, Germany), dorsal hair C57BL/6 mice was completely removed. Mice were then replaced in single cages for 12 h. Testosterone propionate solution (100 μL, 0.5%) in 70% ethanol applied daily for 28 days to dorsal skin to generate AGA-induced mice. Normal control mice were treated with 100 μL of 70% ethanol without testosterone propionate daily, as to not induce AGA. The hair changes on the backs of mice were evaluated daily.

### 4.6. Experimental Design

AGA-induced mice were divided into five treatment groups and assigned the following treatments: (1) 0.1% cellulose in diet (the AGA-Con group; control), (2) 0.1% lyophilized water extract of PL daily in diet (the AGA-PL group), (3) 0.1% lyophilized water extract of PC daily in diet (the AGA-PC group), (4) lyophilized water extracts of 0.05% PL plus 0.05% PC (the AGA-MIX group) daily in a high-fat diet (HFD), or (5) 2 mg finasteride daily application to backs plus 0.1% cellulose in diet (the AGA-Positive group). A total of 2 mg finasteride was used on the back of the AGA-positive mice by applying 100 μL of 2% finasteride solution (2 mg) in 70% ethanol daily. Normal control mice without AGA induction were fed 0.1% cellulose supplementation in HFD (Normal-Con). Mice in each group consumed the designated diet for 28 days. The HFD contained 43% fat (lard and corn oil = 10:1) and 0.5% cholesterol, and 0.1% of the designated herbal extracts or cellulose were added into the HFD. The diets in all groups had equivalent energy and nutrient contents, and control diets contained cellulose, which did not affect AGA symptoms, instead of PL or PC. Food and water were provided ad libitum. Food intakes, body weights, and hair growth statuses were measured weekly.

### 4.7. Assess the Degree of Hair Growth in AGA Mice

Dorsal hair growth was evaluated weekly by assessing hair growth on pictures taken during each evaluation. Hair regrowth ratios (%) were measured blindly by two designated trained technicians. The evaluation criteria for hair regrowth were as follows: score 0 = no growth observed; 1 = up to 20% growth; 2 = 20–40% growth; 3 = 40–60% growth; 4 = 60–80% growth; and 5 = 80% observed full growth [53]

At 0, 1, 2, 3, and 4 weeks, the technicians used tweezers to randomly pluck hair from each group of mice shaved back area and pull at least 30 hairs from each mouse. The hair length was measured using a Vernier caliper (mm). At the same time, 20 hairs were weighed using a precision balance (Sartorius, Germany).

### 4.8. Blood and Tissue Collection and Serum Analysis

On experimental day 28, mice were anesthetized with a ketamine and xylazine mixture (100 and 10 mg/kg body weight, respectively). Serum was separated from venous blood by centrifugation at 500 rpm. Livers and epididymal fat were dissected and weighed using a precision balance (Sartorius, Germany). Dorsal skin tissue was removed and divided into two parts: histopathology or quick-frozen for biochemical assays. Other isolated organs were promptly placed in liquid nitrogen and stored at −70 °C for biochemical experiments.

Total serum testosterone and estradiol were quantified using a Testosterone ELISA Kit and Estradiol ELISA Kit (BD Biosciences, San Diego, CA, USA). Serum total cholesterol and triglyceride and liver tissue triglyceride concentrations were measured using a colorimetric kit (Asan Pharm. Co., Ltd., Seoul, Korea).

### 4.9. Histopathological Analysis

The dorsal skin tissues were fixed in 10% formalin (Sigma-Aldrich, St. Loise, MO, USA) and dehydrated using an ethanol series followed by xylene. Dehydrated back tissues were paraffin-embedded and sectioned at 4 μm using a microtome (Leica Microsystems, Wetzlar, Germany) [54]. Back-skin sections were hematoxylin and eosin-stained (H-E; Sigma-Aldrich, St. Loise, MO, USA) to determine the total hair follicle numbers and their ratio of total skin area and to measure skin thickness using I-Solution software to an optical microscope (Axio Imager 2; Carl Zeiss AG, Oberkochen, Germany; magnification 100×). The dermal thickness ratio was determined with the ratio of the distance between the epidermis and the dermis to the thickness of the total skin layer.

### 4.10. Relative mRNA Expressions of pro-Inflammatory Cytokines, Nuclear Receptors, and Wnt Signaling-Related Genes in Skin Tissues

Total RNA was extracted from dorsal skin tissues using Trizol Reagent (Ambion Inc., Austin, TX, USA), according to the manufacturer’s instructions. cDNA was synthesized from 1 µg of total qualified RNA using the Superscript III reverse transcriptase kit (Bio-Rad, Richmond, CA, USA). During this process, equal amounts of cDNA to total RNA were generated. PCR was conducted using cDNA, primers of genes of interest, and a SYBR Green mixture (Bio-Rad, Richmond, CA, USA). Using Ct values, relative mRNA expressions of genes of interest are determined by real-time fluorescent quantitative PCR (CFX Connect™ real-time PCR detection system; Bio-Rad Laboratories, Inc., Hercules, CA, USA) using Ct values and the 2−ΔΔCt method [55] and β-actin as the housekeeping gene. The primers for the PCR reaction were used as previously described [56].

### 4.11. Western Blot

A frozen skin sample was added into RIPA lysis buffer (1.5, *w*/*v*), and its supernatant was collected after centrifuging at 4 °C at 14,000 rpm for 20 min. The amount of protein in the supernatant was measured using the Bio-Rad protein assay kit (Bio-Rad, Hercules, CA, USA). The lysates having protein (30–50 μg) were resolved into sodium dodecyl sulfate-polyacrylamide gel electrophoresis as described in the previous study [57]. The amount of the interested protein was examined with the specific antibodies as follows: protein kinase B (PKB or Akt), phosphorylated PKB^Ser473^, glycogen synthase kinase (GSK)-3β, and phosphorylated GSK-3β^ser9^. The intensity of the proteins of interest was measured using optical densitometry (I-Solution software).

### 4.12. Statistical Analysis

Statistical analysis was performed using SPSS version 20.0 (IBM Corp., Armonk, NY, USA). Results are presented as means ± standard deviations or frequency distributions. A one-way analysis of variance (ANOVA) was used to determine the significances of intergroup differences, and if its result was significant, Tukey’s test was used for multiple comparisons between groups. Statistical significance was accepted for *p* values < 0.05.

## Figures and Tables

**Table 1:** Body composition and food intake at the end of the experimental periods.

	AGA-Con (N = 12)	AGA-Positive (N = 12)	AGA-PL (N = 12)	AGA- PC (N = 12)	AGA-MIX (N = 11)	Normal-Con (N = 11)
Final weight (g)	25.6 ± 0.76	26.8 ± 0.55	26.7 ± 0.58	25.4 ± 0.69	25.5 ± 0.39	26.5 ± 0.38
Weight gain (g/8 weeks)	3.71 ± 0.32 ^ab^	4.13 ± 0.38 ^a^	3.83 ± 0.39 ^ab^	2.64 ± 0.38 ^b^	2.55 ± 0.42 ^b^	3.25 ± 0.31 ^ab^
Food intake (g/day)	4.10 ± 0.31	3.59 ± 0.20	4.08 ± 0.33	3.48 ± 0.40	3.78 ± 0.27	3.22 ± 0.28
PL or PC intake (mg/kg bw/day)	-	-	153 ± 12	139 ± 16	148 ± 11	-
Efficiency of food	0.97 ± 0.10 ^ab^	1.14 ± 0.07 ^a^	0.94 ± 0.08 ^ab^	0.76 ± 0.13 ^ab^	0.67 ± 0.09 ^b^	1.09 ± 0.12 ^a^
Epididymal fat (g)	0.53 ± 0.06 ^b^	0.68 ± 0.06 ^a^	0.55 ± 0.07 ^b^	0.49 ± 0.04 ^b^	0.40 ± 0.03 ^c^	0.59 ± 0.04 ^b^
Retroperitoneal fat (g)	0.18 ± 0.02	0.25 ± 0.02	0.20 ± 0.04	0.19 ± 0.02	0.15 ± 0.02	0.22 ± 0.02
Total visceral fat (g)	0.72 ± 0.07 ^b^	0.93 ± 0.09 ^a^	0.75 ± 0.11 ^b^	0.67 ± 0.06 ^b^	0.55 ± 0.04 ^c^	0.81 ± 0.05 ^b^

AGA was induced with the application of 0.5% testosterone propionate solution on the back of mice, and AGA-induced mice orally consumed 0.1% PL, 0.1% PC, or 0.1% mixture of PL and PC (1:1, *w*/*w*) in a 43% fat diet (HFD), called AGA-PL, AGA-PC, or AGA-MIX groups, respectively. Among, AGA-induced mice, 2% finasteride was applied daily to the back, as AGA-Positive (positive control). AGA-Positive and AGA-Con (control) mice had 0.1% cellulose (no effective activity in AGA treatment) in an HFD. Normal-Con did not induce AGA and consumed 0.1% cellulose in an HFD. All mice had an 8-week treatment. The efficiency of food was obtained by dividing the weight gain by the food intake. Values are means ± standard deviations. ^a,b,c^ Different superscript letters on the means indicate a significant difference as per a Tukey test at *p* < 0.05, and no or the same letters indicate no significance between the groups.

**Table 2:** Sex hormones, cholesterol, and triglyceride concentrations in the circulation.

	AGA-Con (N = 12)	AGA-Positive (N = 12)	AGA-PL (N = 12)	AGA- PC (N = 12)	AGA-MIX (N = 11)	Normal-Con (N = 11)
Serum testosterone (ng/mL)	27.10 ± 0.81 ^a^	27.83 ± 0.62 ^a^	21.15 ± 0.78 ^b^	23.88 ± 0.38 ^ab^	24.54 ± 0.56 ^ab^	21.23 ± 1.33 ^b^
Serum 17β-estradiol (ng/mL)	13.95 ± 2.01 ^b^	16.14 ± 1.86 ^ab^	19.38 ± 0.86 ^a^	17.69 ± 1.84 ^ab^	16.78 ± 1.39 ^ab^	18.71 ± 1.86 ^a^
Serum total cholesterol (mg/dL)	149.39 ± 6.71	149.96 ± 8.72	137.10 ± 5.04	135.53 ± 2.99	137.28 ± 4.35	144.98 ± 4.20
Serum triglyceride (mg/dL)	63.68 ± 4.96 ^a^	64.19 ± 4.15 ^a^	47.51 ± 3.48 ^ab^	34.64 ± 2.86 ^b^	58.06 ± 2.80 ^a^	46.65 ± 2.67 ^ab^

AGA was induced with the application of 0.5% testosterone propionate solution on the back of mice, and AGA-induced mice orally consumed 0.1% PL, 0.1% PC, or 0.1% mixture of PL and PC (1:1, *w*/*w*) in a 43% fat diet (HFD), called AGA-PL, AGA-PC, or AGA-MIX groups, respectively. AGA-induced mice were daily applied with 2% finasteride on the back, as AGA-Positive (positive control). AGA-Positive and AGA-Con (control) mice had 0.1% cellulose (no effective activity in AGA treatment) in an HFD. Normal-Con did not induce AGA and consumed 0.1% cellulose in an HFD. All mice had an 8-week treatment. Values are means ± standard deviations. ^a,b^ Different superscripts on the means indicate a significant difference as per a Tukey test at *p* < 0.05, and no or the same superscripts included on them indicate no significance between the groups.

## Data Availability

Data is contained within the article and Supplementary Materials.

## Supplementary Materials

https://www.mdpi.com/article/10.3390/ph14111128/s1. Table S1. Active ingredients of Paeonia lactiflora Pallas (PL) and Poria cocos Wolf (PC) from Traditional Chinese Medicine system pharmacology database and analysis platform. Figure S1 HPLC chromatogram of *Paeonia lactiflora pallas* and *Poria cocos Wolf* water extracts. Figure S2. Protein-protein interaction network construction diagram for PL, PC and androgenetic alopecia (AGA).

## Abbreviations

PL, *Paeonia lactiflora* Pallas*;* PC, *Poria cocos* Wolf*;* AGA, androgenetic alopecia; DKK1, dickkopf-related protein 1; NR3C2, nuclear receptor subfamily 3 group; PPARG, peroxisome proliferator-activated receptor-gamma; β-Catenin, catenin beta 1; TGF-β2, Tumor growth factor-beta 2; TNF-α, tumor necrosis factor-α; IL-6, interleukin 6; CYP19A1(Aromatase), Cytochrome P450 Family 19 Subfamily A Member 1; TGF-β2, Transforming Growth Factor-Beta 2; DHT, dihydrotestosterone; FDA, food and drug administration; EMA, European medicines agency; TCM, traditional Chinese medicine; TCMSP, traditional Chinese medicine systems pharmacology database and analysis platform; ADME, absorption distribution metabolism and excretion; OB, oral bioavailability; DL, drug-likeness; PPI, protein–protein interaction; GO, gene ontology; BW, body weight; PCR, polymerase chain reaction; SD, standard deviation; ANOVA, a one-way analysis of variance.

## References

[1] Nanes B.A. (2020). Androgenetic alopecia in COVID-19: Compared to what?. J. Am. Acad. Dermatol..

[2] Zheng Y., Hu Y., Liu K., Lu Y., Hu Y., Zhou X. (2019). Therapeutic effect of Impatiens balsamina, Lawsonia inermis L. and Henna on androgenetic alopecia in mice. J. South. Med Univ..

[3] Dhariwala M.Y., Ravikumar P. (2019). An overview of herbal alternatives in androgenetic alopecia. J. Cosmet. Dermatol..

[4] Lolli F., Pallotti F., Rossi A., Fortuna M.C., Caro G., Lenzi A., Sansone A., Lombardo F. (2017). Androgenetic alopecia: A review. Endocrine.

[5] Sadgrove N.J. (2021). The ‘Bald’ Phenotype (Androgenetic Alopecia) is Caused by the High Glycaemic, High Cholesterol and Low Mineral ‘Western Diet’. Trends Food Sci. Technol..

[6] Lee S.W., Juhasz M., Mobasher P., Ekelem C., Mesinkovska N.A. (2018). A Systematic Review of Topical Finasteride in the Treatment of Androgenetic Alopecia in Men and Women. J. Drugs Dermatol..

[7] Mysore V., Shashikumar B.M. (2016). Guidelines on the use of finasteride in androgenetic alopecia. Indian J. Dermatol. Venereol. Leprol..

[8] Almohanna H.M., Perper M., Tosti A. (2018). Safety concerns when using novel medications to treat alopecia. Expert Opin. Drug Saf..

[9] Suchonwanit P., Iamsumang W., Leerunyakul K. (2020). Topical finasteride for the treatment of male androgenetic alopecia and female pattern hair loss: A review of the current literature. J. Dermatol. Treat..

[10] Piraccini B.M., Blume-Peytavi U., Scarci F., Jansat J.M., Falqués M., Otero R., Tamarit M.L., Galván J., Tebbs V., Massana E. (2021). Efficacy and safety of topical finasteride spray solution for male androgenetic alopecia: A phase III, randomized, controlled clinical trial. J. Eur. Acad. Dermatol. Venereol. JEADV.

[11] Monti D., Tampucci S., Burgalassi S., Chetoni P., Lenzi C., Pirone A., Mailland F. (2014). Topical formulations containing finasteride. Part I: In vitro permeation/penetration study and in vivo pharmacokinetics in hairless rat. J. Pharm. Sci..

[12] Noubarani M., Rostamkhani H., Erfan M., Kamalinejad M., Eskandari M.R., Babaeian M., Salamzadeh J. (2014). Effect of Adiantum Capillus veneris Linn on an Animal Model of Testosterone-Induced Hair Loss. Iran. J. Pharm. Res. IJPR.

[13] Sadgrove N.J. (2018). The new paradigm for androgenetic alopecia and plant-based folk remedies: 5α-reductase inhibition, reversal of secondary microinflammation and improving insulin resistance. J. Ethnopharmacol..

[14] Mao X., Chen W.J., Li Y.F., Li W.J., Li T.X., Wang X.Y., Guo M.Q., Zhang Y.Q., Lin N. (2019). An exploration into the therapeutic effects and molecular mechanisms of paeoniflorin in the treatment of adjuvant-induced arthritis rats by a network pharmacology-based research strategy. Acta Pharm..

[15] Parker S., May B., Zhang C., Zhang A.L., Lu C., Xue C.C. (2016). A Pharmacological Review of Bioactive Constituents of Paeonia lactiflora Pallas and Paeonia veitchii Lynch. Phytother Res..

[16] He D.Y., Dai S.M. (2011). Anti-inflammatory and immunomodulatory effects of paeonia lactiflora pall., a traditional chinese herbal medicine. Front. Pharmacol..

[17] Liu J., Jin D.Z., Xiao L., Zhu X.Z. (2006). Paeoniflorin attenuates chronic cerebral hypoperfusion-induced learning dysfunction and brain damage in rats. Brain Res..

[18] Jia X., Ma L., Li P., Chen M., He C. (2016). Prospects of Poria cocos polysaccharides: Isolation process, structural features and bioactivities. Trends Food Sci. Technol..

[19] Feng Y.L., Lei P., Tian T., Yin L., Chen D.Q., Chen H., Mei Q., Zhao Y.Y., Lin R.C. (2013). Diuretic activity of some fractions of the epidermis of Poria cocos. J. Ethnopharmacol..

[20] Sun S.S., Wang K., Ma K., Bao L., Liu H.W. (2019). An insoluble polysaccharide from the sclerotium of Poria cocos improves hyperglycemia, hyperlipidemia and hepatic steatosis in ob/ob mice via modulation of gut microbiota. Chin. J. Nat. Med..

[21] Ríos J.L. (2011). Chemical constituents and pharmacological properties of Poria cocos. Planta Med..

[22] Goren A., Cadegiani F.A., Wambier C.G., Vano-Galvan S., Tosti A., Shapiro J., Mesinkovska N.A., Ramos P.M., Sinclair R., Lupi O. (2021). Androgenetic alopecia may be associated with weaker COVID-19 T-cell immune response: An insight into a potential COVID-19 vaccine booster. Med. Hypotheses.

[23] Ru J., Li P., Wang J., Zhou W., Li B., Huang C., Li P., Guo Z., Tao W., Yang Y. (2014). TCMSP: A database of systems pharmacology for drug discovery from herbal medicines. J. Cheminform..

[24] Kim J.H., Na J., Bak D.H., Lee B.C., Lee E., Choi M.J., Ryu C.H., Lee S., Mun S.K., Park B.C. (2019). Development of finasteride polymer microspheres for systemic application in androgenic alopecia. Int. J. Mol. Med..

[25] Wang Z.-D., Feng Y., Ma L.-Y., Li X., Ding W.-F., Chen X.-M. (2017). Hair growth promoting effect of white wax and policosanol from white wax on the mouse model of testosterone-induced hair loss. Biomed. Pharmacother..

[26] Orăsan M.S., Coneac A. (2017). Evaluation of Animal Models Suitable for Hair Research and Regeneration, Experimental Animal Models of Human Diseases—An Effective Therapeutic Strategy.

[27] Lee S.Y., Lee D.J., Kwon K., Lee C.H., Shin H.J., Kim J.E., Ha K.T., Jeong H.S., Seo H.S. (2016). Cornu cervi pantotrichum Pharmacopuncture Solution Facilitate Hair Growth in C57BL/6 Mice. J. Pharmacopunct..

[28] Gang L., Bo X., Liang X.-Z., Gai S.-S., Xia C.-M., Yan B.-Z., Li J.-C. (2018). Study on the molecular mechanism of osteoporosis treated by Epimedium based on network pharmacology. Chin. Pharmacol. Bull..

[29] Fu D., Huang J., Li K., Chen Y., He Y., Sun Y., Guo Y., Du L., Qu Q., Miao Y. (2021). Dihydrotestosterone-induced hair regrowth inhibition by activating androgen receptor in C57BL6 mice simulates androgenetic alopecia. Biomed Pharm..

[30] English R.S. (2018). A hypothetical pathogenesis model for androgenic alopecia: Clarifying the dihydrotestosterone paradox and rate-limiting recovery factors. Med. Hypotheses.

[31] Truong V.L., Bak M.J., Lee C., Jun M., Jeong W.S. (2017). Hair Regenerative Mechanisms of Red Ginseng Oil and Its Major Components in the Testosterone-Induced Delay of Anagen Entry in C57BL/6 Mice. Molecules.

[32] Grymowicz M., Rudnicka E., Podfigurna A., Napierala P., Smolarczyk R., Smolarczyk K., Meczekalski B. (2020). Hormonal Effects on Hair Follicles. Int. J. Mol. Sci..

[33] Sun Y. (2014). Biological activities and potential health benefits of polysaccharides from Poria cocos and their derivatives. Int. J. Biol. Macromol..

[34] Chang Y., Wei W., Zhang L., Xu H.-M. (2009). Effects and mechanisms of total glucosides of paeony on synoviocytes activities in rat collagen-induced arthritis. J. Ethnopharmacol..

[35] Ong M., Cheng J., Jin X., Lao W., Johnson M., Tan Y., Qu X. (2019). Paeoniflorin extract reverses dexamethasone-induced testosterone over-secretion through downregulation of cytochrome P450 17A1 expression in primary murine theca cells. J. Ethnopharmacol..

[36] Li S., Zhang B., Zhang N. (2011). Network target for screening synergistic drug combinations with application to traditional Chinese medicine. BMC Syst. Biol..

[37] Li P., Su W. (2016). Recent progress in applying network pharmacology to research of Chinese materia medica. Chin. Tradit. Herb. Drugs.

[38] Ansari S., Bari A., Ullah R., Mathanmohun M., Veeraraghavan V.P., Sun Z. (2019). Gold nanoparticles synthesized with Smilax glabra rhizome modulates the anti-obesity parameters in high-fat diet and streptozotocin induced obese diabetes rat model. J. Photochem. Photobiol. B Biol..

[39] Zhao J., Niu X., Yu J., Xiao X., Li W., Zang L., Hu Z., Siu-Po Ip P., Li W. (2020). Poria cocos polysaccharides attenuated ox-LDL-induced inflammation and oxidative stress via ERK activated Nrf2/HO-1 signaling pathway and inhibited foam cell formation in VSMCs. Int. Immunopharmacol..

[40] Zhang Y., Xu J., Jing J., Wu X., Lv Z. (2018). Serum Levels of Androgen-Associated Hormones Are Correlated with Curative Effect in Androgenic Alopecia in Young Men. Med. Sci. Monit..

[41] Chen X., Liu B., Li Y., Han L., Tang X., Deng W., Lai W., Wan M. (2020). Dihydrotestosterone Regulates Hair Growth Through the Wnt/β-Catenin Pathway in C57BL/6 Mice and In Vitro Organ Culture. Front. Pharmacol..

[42] Peyravian N., Deo S., Daunert S., Jimenez J.J. (2020). The Inflammatory Aspect of Male and Female Pattern Hair Loss. J. Inflamm. Res..

[43] Liu J., Yu J., Peng X. (2020). Poria cocos Polysaccharides Alleviates Chronic Nonbacterial Prostatitis by Preventing Oxidative Stress, Regulating Hormone Production, Modifying Gut Microbiota, and Remodeling the DNA Methylome. J. Agric. Food Chem..

[44] Wang Q.S., Gao T., Cui Y.L., Gao L.N., Jiang H.L. (2014). Comparative studies of paeoniflorin and albiflorin from Paeonia lactiflora on anti-inflammatory activities. Pharm. Biol..

[45] Andy G., John M., Mirna S., Rachita D., Michael K., Maja K., Aseem S., Zeljana B. (2019). Controversies in the treatment of androgenetic alopecia: The history of finasteride. Dermatol. Ther..

[46] Duborija-Kovacevic N., Jakovljevic V., Sabo A., Tomic Z. (2008). Anti-nociceptive and anti-inflammatory properties of 5alpha-reductase inhibitor finasteride in experimental animals. Eur. J. Drug Metab. Pharmacokinet..

[47] http://tcmspw.com/tcmsp.php.

[48] Https://www.genecards.org/.

[49] Https://omim.org/.

[50] Https://www.malacards.org/.

[51] Https://string-db.org/.

[52] Http://www.bioconductor.org/.

[53] Zhang B., Zhang R.W., Yin X.Q., Lao Z.Z., Zhang Z., Wu Q.G., Yu L.W., Lai X.P., Wan Y.H., Li G. (2016). Inhibitory activities of some traditional Chinese herbs against testosterone 5α-reductase and effects of Cacumen platycladi on hair re-growth in testosterone-treated mice. J. Ethnopharmacol..

[54] Jeong S.Y., Im Y.N., Youm J.Y., Lee H.K., Im S.Y. (2018). l-Glutamine Attenuates DSS-Induced Colitis via Induction of MAPK Phosphatase-1. Nutrients.

[55] Livak K.J., Schmittgen T.D. (2001). Analysis of relative gene expression data using real-time quantitative PCR and the 2(-Delta Delta C(T)) Method. Methods.

[56] Ellis J.A., Sinclair R., Harrap S.B. (2002). Androgenetic alopecia: Pathogenesis and potential for therapy. Expert Rev. Mol. Med..

[57] Kim D.S., Ko B.-S., Ryuk J.A., Park S. (2020). Tetragonia tetragonioides Protected against Memory Dysfunction by Elevating Hippocampal Amyloid-β Deposition through Potentiating Insulin Signaling and Altering Gut Microbiome Composition. Int. J. Mol. Sci..

