# Supplementary material for: Alleviation of Androgenetic Alopecia with Aqueous Paeonia lactiflora and Poria cocos Extract Intake through Suppressing the Steroid Hormone and Inflammatory Pathway
Source: Pharmaceuticals (Basel). 2021 Nov 5;14(11):1128. doi: 10.3390/ph14111128 (PMC8621879; doi:10.3390/ph14111128)
Supplement: Supplementary file 1 [file pharmaceuticals-14-01128-s001.zip › pharmaceuticals-1423058-supplementary.pdf]

Table S1. Active ingredients of *Paeonia lactiflora* Pallas (PL) and *Poria cocos* Wolf (PC) from Traditional Chinese Medicine system pharmacology database and analysis platform

**Molecular ID**	**Molecular name**	**OB (%)**	**DL**	**Herb**
MOL001910	11alpha,12alpha-epoxy-3beta-23-dihydroxy-30-norolean-20-en-28,12beta-olide	64.77	0.38	PL
MOL001918	paeoniflorgenone	87.59	0.37	PL
MOL001919	(3S,5R,8R,9R,10S,14S)-3,17-dihydroxy-4,4,8,10,14-pentamethyl-2,3,5,6,7,9-hexahydro-1H-cyclopenta[a]phenanthrene-15,16-dione	43.56	0.53	PL
MOL001921	lactiflorin	49.12	0.8	PL
MOL001924	paeoniflorin	53.87	0.79	PL
MOL001925	paeoniflorin_qt	68.18	0.4	PL
MOL001928	albiflorin_qt	66.64	0.33	PL
MOL001930	benzoyl paeoniflorin	31.27	0.75	PL
MOL000211	mairin	55.38	0.78	PL
MOL000358	beta-sitosterol	36.91	0.75	PL
MOL000359	sitosterol	36.91	0.75	PL
MOL000422	kaempferol	41.88	0.24	PL
MOL000492	(+)-catechin	54.83	0.24	PL
MOL000273	(2R)-2-[(3S,5R,10S,13R,14R,16R,17R)-3,16-dihydroxy-4,4,10,13,14-pentamethyl-2,3,5,6,12,15,16,17-octahydro-1H-cyclopenta[a]phenanthren-17-yl]-6-methylhept-5-enoic acid	30.93	0.81	PC
MOL000275	trametenolic acid	38.71	0.80	PC
MOL000276	7,9(11)-dehydropachymic acid	35.11	0.81	PC
MOL000279	cerevisterol	37.96	0.77	PC
MOL000280	(2R)-2-[(3S,5R,10S,13R,14R,16R,17R)-3,16-dihydroxy-4,4,10,13,14-pentamethyl-2,3,5,6,12,15,16,17-octahydro-1H-cyclopenta[a]phenanthren-17-yl]-5-isopropyl-hex-5-enoic acid	31.07	0.82	PC
MOL000282	ergosta-7,22E-dien-3beta-ol	43.51	0.72	PC
MOL000283	ergosterol peroxide	40.36	0.81	PC
MOL000285	(2R)-2-[(5R,10S,13R,14R,16R,17R)-16-hydroxy-3-keto-4,4,10,13,14-pentamethyl-1,2,5,6,12,15,16,17-octahydrocyclopenta[a]phenanthren-17-yl]-5-isopropyl-hex-5-enoic acid	38.26	0.82	PC

MOL000289	pachymic acid	33.63	0.81	PC
MOL000290	poricoic acid A	30.61	0.76	PC
MOL000291	poricoic acid B	30.52	0.75	PC
MOL000292	poricoic acid C	38.15	0.75	PC
MOL000296	hederagenin	36.91	0.75	PC
MOL000300	dehydroeburicoic acid	44.17	0.83	PC
MOL000287	3beta-Hydroxy-24-methylene-8-lanostene-21-oic acid	38.7	0.81	PC

OB, oral bioavailability; DL, drug-likeness

The higher OB and DL were the better activity as a drug.

Figure S1. Protein-protein interaction network construction diagram for PL, PC and androgenetic alopecia (AGA).

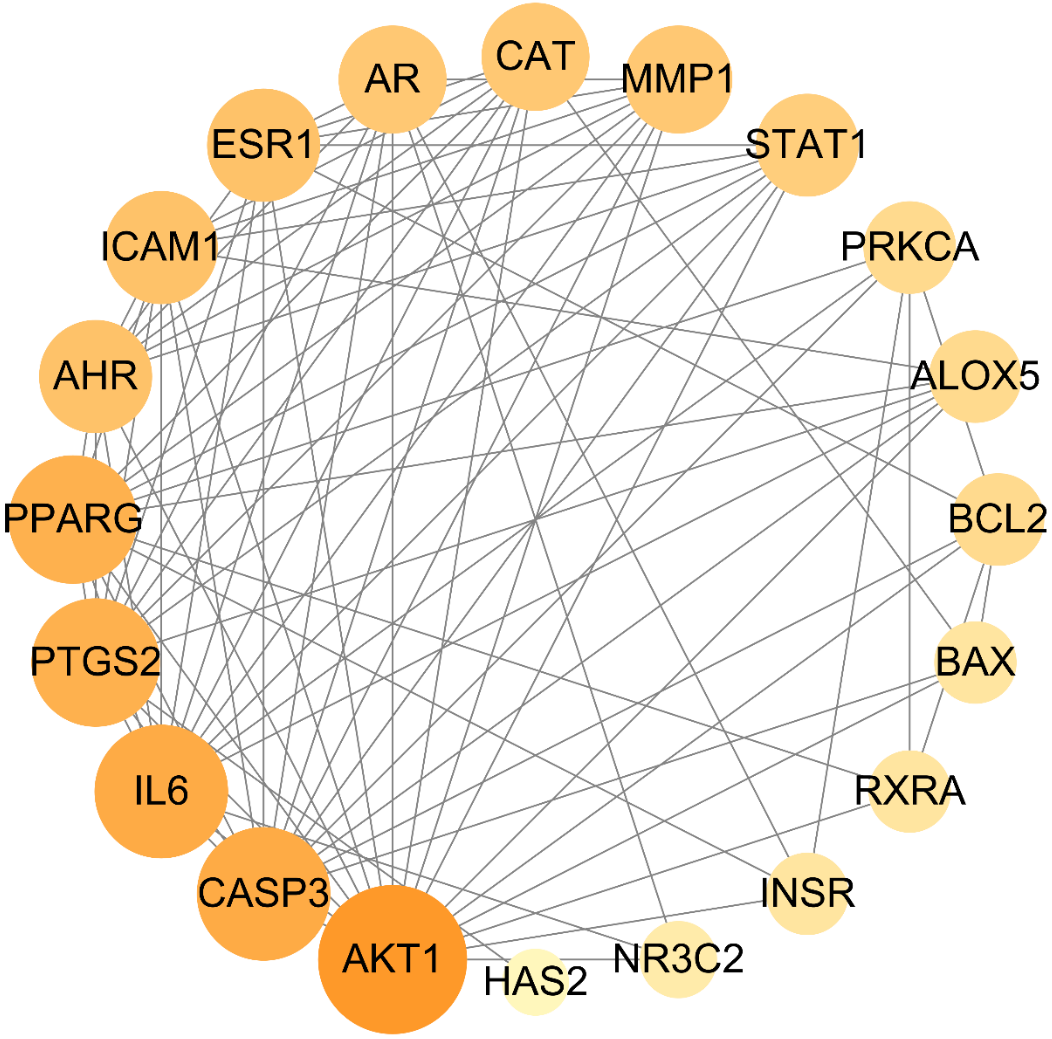

Each circle represents a protein, and lines between proteins represent relationships.

The Larger circles and darker colors were the more related proteins to the AGA and active components.

Figure S2. HPLC chromatogram of *Paeonia lactiflora* pallas and *Poria cocos* Wolf water extracts

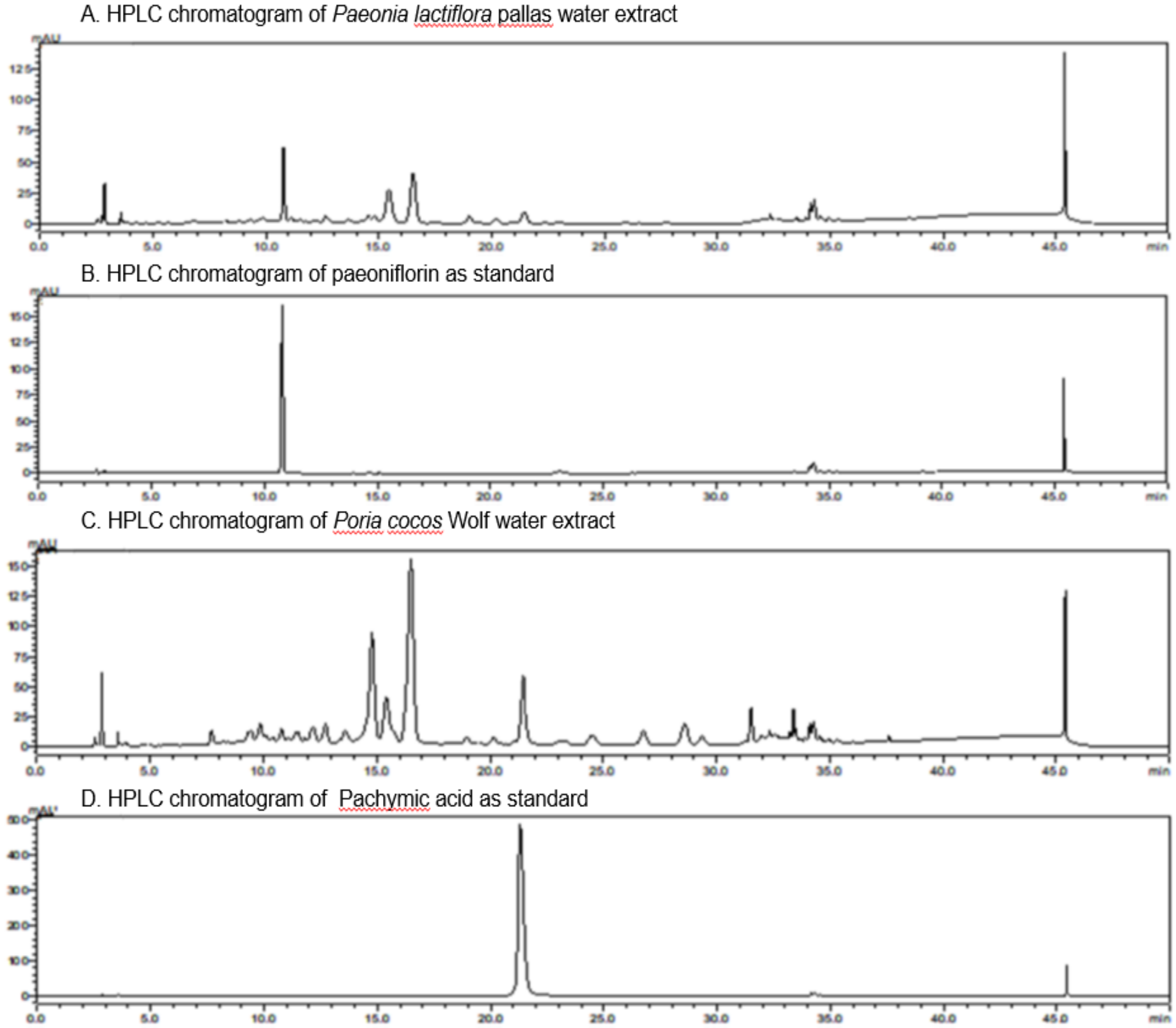

The concentrations of paeoniflorin and pachymic acid were 1 mg/mL in methanol, as stock to dilute them according to the sample concentrations with methanol. HPLC analysis was performed using an Agilent 1100 series HPLC system (Agilent Technologies, Waldbronn, Germany) composed of a quaternary pump (G1315B), vacuum degasser (G1379A), column oven (G1316A), diode-array detector (G1315B), and autosampler (G1313A). Chromatographic data were processed using the ChemStation software. Chromatographic separation was performed using a XD8 C18 column (4.6 mm × 150 mm; inner diameter: 5 μm). The column oven was maintained at 30°C and detection was performed at λ = 233 nm for paeoniflorin and 235 nm for pachmic acid. The mobile gradient phase for paeoniflorin was 90:10 → 78:2 for 25 min → 20: 80 for 25 min of distilled water/ acetonitrile while that for pachymic acid was 30:70 → 100% for 25 min → 100% water/acetonitrile with 1% acetic acid for 25 min. The flow rate was 1.0 mL/min and the injection volume was 10 μL.
